# Medication adherence in Jordanian patients with multimorbidity: a cross-sectional mixed-methods study in outpatient clinics

**DOI:** 10.3389/fphar.2025.1619023

**Published:** 2025-07-31

**Authors:** Anas Abed, Mohammad Abu Assab, Wael Abu Dayyih, Badriyah S. Alotaibi, Nawal Alsubaie

**Affiliations:** ^1^ Department of Biopharmaceutics and Clinical Pharmacy, Faculty of Pharmacy, Al-Ahliyya Amman University, Amman, Jordan; ^2^ Clinical Pharmacy Department, Faculty of Pharmacy, Zarqa University, Zarqa, Jordan; ^3^ Faculty of Pharmacy, Mutah University, Alkarak, Jordan; ^4^ Department of Pharmaceutical Sciences, College of Pharmacy, Princess Nourah bint Abdulrahman University, P.O. Box 84428, Riyadh 11671, Saudi Arabia; ^5^ Department of Pharmacy Practice, College of Pharmacy, Princess Nourah bint Abdulrahman University, P.O. Box 84428, Riyadh 11671, Saudi Arabia

**Keywords:** medication adherence, multimorbidity, chronic disease multimorbidity, polypharmacy, mixed-methods approach

## Abstract

**Background:**

Multimorbidity, the coexistence of two or more chronic conditions, is increasingly prevalent in Jordan, a middle-income country with a growing non-communicable disease (NCD) burden and limited data on medication adherence. This study examined adherence prevalence, predictors, and barriers among Jordanians with multimorbidity to inform interventions supporting Sustainable Development Goal 3.

**Methods:**

A cross-sectional, mixed-methods study was conducted from April to December 2024 in two tertiary Hospitals’ outpatient clinics in Amman. Adults (≥18 years) with ≥2 chronic conditions requiring ongoing medication (*n* = 405) were recruited via convenience sampling. Adherence was assessed using the General Medication Adherence Scale (GMAS, 0–33; high adherence defined as GMAS ≥27) alongside a Self-Administered Comorbidity Questionnaire (SCQ)-like checklist. Qualitative data were collected through an open-ended question and analyzed using thematic analysis. Descriptive statistics, correlation analysis, and logistic regression were used. Qualitative data were analysed using thematic analysis.

**Results:**

Of 420 invited participants, 405 completed the survey (response rate: 96.4%). High adherence (GMAS ≥27) was observed in 54.3% of participants. Factors positively associated with medication adherence included older age, higher income, fewer medications, higher health literacy, and social support, while cost, polypharmacy, forgetfulness, rural residence, and low perceived medication necessity were key barriers. Six qualitative themes were identified: economic strain, access gaps, cultural beliefs, religious coping, caregiving burden, and symptom management issues.

**Conclusion:**

This first study of medication adherence in Jordanian multimorbidity highlights actionable barriers and facilitators, offering a scalable model for resource-limited settings. Subsidies, mobile pharmacies, and culturally tailored strategies could substantially reduce the non-adherence rates, advancing NCD control regionally and globally.

## 1 Introduction

Multimorbidity, the coexistence of two or more chronic conditions in an individual, is a growing global health challenge, particularly in aging populations and rapidly developing economies (Skou et al., 2022). Recent estimates indicate that approximately one-third of adults worldwide have at least two chronic diseases (Chowdhury et al., 2023). Driven by demographic transitions, population aging, and the escalating burden of non-communicable diseases (NCDs) such as diabetes, hypertension, and cardiovascular disease, multimorbidity now affects approximately 23%–33% of adults in community settings and rises to over 75% among those aged 70 years and older (Richards et al., 2016; Nguyen et al., 2019; Singh et al., 2022). Projections suggest a steep increase by 2035, particularly in low- and middle-income countries (LMICs), where health systems often remain ill-equipped to manage complex, long-term care needs efficiently (Kingston et al., 2018).

Medication adherence—the degree to which patients take their medications as prescribed- is central to the effective management of multimorbidity. High adherence is vital for achieving disease control, preventing complications, and reducing healthcare utilization (Religioni et al., 2025). For example, a meta-analysis of older adults found that good adherence was associated with a ∼21% reduction in long-term mortality risk compared to medication non-adherence (Walsh et al., 2019). Conversely, poor adherence leads to suboptimal disease control, higher hospitalization rates, and increased mortality (Neiman et al., 2018; Walsh et al., 2019). In other words, patients managing multiple chronic conditions derive substantial benefit from medication adherence, whereas lapses in medication-taking can rapidly compound morbidity and mortality risks.

Medication non-adherence also carries a tremendous economic burden for health systems. In the United States, it is estimated that poor adherence contributes to approximately $100–300 billion in avoidable healthcare costs annually (Iuga and McGuire, 2014; Cutler et al., 2018; Kini and Ho, 2018). This includes costs from preventable hospitalizations, emergency visits, and disease complications attributable to patients not following treatment plans. Globally, the economic impact is also alarming. In Europe, for instance, medication non-adherence is associated with around 200,000 premature deaths and €80–125 billion in health expenditures per year (Horvat et al., 2024).

Multiple factors contribute to poor adherence, especially in patients with multimorbidity who often face complex treatment regimens. Patients with multimorbidity face significant adherence barriers due to psychological and social factors (Zakaraya et al., 2023). Polypharmacy–the use of multiple medications–can impose a high treatment burden, leading to unintentional non-adherence, such as forgetting doses or confusion about schedules (Liu et al., 2023). In addition to complicating adherence, polypharmacy increases the risk of adverse drug events, drug-drug interactions, and medication errors, highlighting the pharmacological importance of adherence interventions in patients with multimorbidity (Maher et al., 2014).

Intentional medication non-adherence is another challenge; some patients may skip medications due to perceived lack of efficacy, fear of side effects, or cultural beliefs and misconceptions about their illnesses (Kvarnström et al., 2021). Critically, financial barriers can strongly influence medication adherence in low-resource settings. Many patients in LMICs struggle with the out-of-pocket costs of medications. For example, among Syrian refugees with NCDs in Jordan, 61.5% cited medication cost as the main barrier to obtaining care (Rehr et al., 2018). Similarly, inadequate insurance coverage contributes to medication non-adherence (Panahi et al., 2022). These financial obstacles, combined with limited health literacy and healthcare access gaps, create a multifaceted adherence problem–particularly for multimorbid patients who must navigate multiple co-existing illnesses and treatments simultaneously. To conceptually frame these complexities, this study is guided by the World Health Organization’s Five Dimensions of Adherence, which categorize determinants into health system, socioeconomic, condition-related, therapy-related, and patient-related factors, underscoring the need for holistic analysis and intervention (WHO, 2003).

Despite the recognized importance of adherence to therapeutic drugs in chronic disease outcomes, there is a notable data gap in multimorbidity-specific medication adherence research in the Middle East, including Jordan. Most studies in the region to date have focused on single diseases (e.g., diabetes or hypertension) when examining adherence behaviors, rather than on patients managing multiple concurrent conditions. Consequently, little is known about how medication adherence challenges manifest in patients with multimorbidity in this context. In Jordan, research on medication adherence among multimorbid populations is extremely limited, leaving health authorities with minimal local evidence to guide interventions. This gap in knowledge hinders the development of effective, targeted strategies to improve medication adherence and health outcomes for patients with multiple chronic conditions.

To address this gap, we conducted a study examining medication adherence among Jordanians with multimorbidity attending two outpatient clinics at tertiary hospitals in Amman. Using the General Medication Adherence Scale (GMAS) and a tailored comorbidity checklist adapted from the Self-Administered Comorbidity Questionnaire (SCQ), we quantified adherence prevalence and explored its sociodemographic, clinical, and economic predictors while capturing patient-reported barriers and facilitators. A mixed-methods approach was chosen to complement these quantitative findings with qualitative insights into patient experiences, enabling a more comprehensive understanding of adherence behaviors within the local sociocultural and healthcare context. By situating our findings within Jordan’s healthcare system and the global Sustainable Development Goals framework, we aim to guide targeted interventions, enhance therapeutic outcomes, and contribute to the sparse literature on multimorbidity in the Eastern Mediterranean region.

## 2 Materials and methods

### 2.1 Study design and setting

This cross-sectional study was conducted from April to December 2024 to evaluate medication adherence among Jordanians with multimorbidity, defined as having two or more chronic conditions requiring ongoing medication management. The research aimed to identify barriers and facilitators to medication adherence, integrating quantitative and qualitative data to guide targeted interventions.

Data collection took place at two outpatient clinics at two tertiary hospitals in Amman offering comprehensive chronic disease management. This multi-centre approach streamlined recruitment and utilized the diverse patient population, including both urban and rural referrals over the 9-month period.

### 2.2 Participants

The study population comprised adults aged 18 years and older with multimorbidity, attending routine outpatient visits at the outpatient clinics. Eligibility required a confirmed diagnosis of at least two chronic conditions by a healthcare provider and current use of multiple prescription medications, excluding individuals unable to provide consent due to cognitive or language barriers.

### 2.3 Sample size calculation

The sample size was calculated to detect a significant difference in adherence rates, assuming a baseline high medication adherence rate of 50% (based on regional estimates) and aiming to identify a 15% difference between groups defined by key predictors. With a power of 80%, a two-sided alpha of 0.05, and an expected effect size of a 15% difference in adherence rates between groups, a minimum of 376 participants was required, calculated based standard formulas for comparing proportions. Accounting for a 10% non-response rate, the target was adjusted to approximately 420 participants, with 405 achieved, deemed sufficient for robust statistical analysis. Participants were recruited using a convenience sampling strategy by patients in the waiting areas during their scheduled follow-ups.

### 2.4 Data collection and measurement tools

Data were collected via a structured, self-administered questionnaire in Arabic, distributed in the clinic’s waiting areas with each survey taking 15–20 min. Sociodemographic data (age, sex, education, income, insurance, residence) and clinical details (conditions, medications) were recorded, cross-verified with clinic records where feasible. Clinical details were gathered using a patient-reported checklist of 13 chronic conditions, adapted from the Self-Administered Comorbidity Questionnaire (SCQ), asking participants to indicate presence, treatment status, activity limitation, and control of each condition. Where feasible, responses were cross-verified with clinic records to enhance accuracy, and prescribed medications were similarly recorded.

Medication adherence was assessed using the General Medication adherence Scale (GMAS), an 11-item tool scoring 0–33 across three domains: patient behavior (4 items), pill burden (4 items), and cost-related non-adherence (3 items). Responses used a Likert-type scale (always to never), with categories of high (30–33), good (27–29), partial (11–26), and low (≤10), and a binary split at ≥27 versus ≤26.

The Arabic version of the GMAS, previously validated in Saudi chronic diseases populations (Naqvi et al., 2020), was used without modification. The GMAS showed good reliability (Cronbach’s alpha = 0.8705).

Pill burden was defined as the total number of daily medications, captured through participant reports and verified with prescription records, with higher counts indicating greater burden.

In addition to the structured questionnaire, two open-ended questions were administered to explore participants’ perspectives on medication adherence:

(1) *“What challenges do you face in taking your medications as prescribed?”*
(2) *“What helps you take your medications regularly?”*

These were presented after a set of multiple-choice barrier/facilitator options to prompt reflection, including an “Other (please specify)” field that allowed participants to elaborate in their own words.

To ensure inclusivity across literacy levels, trained bilingual interviewers (Arabic/English) assisted participants in clinic waiting areas, effectively mitigating literacy-related bias in data collection. For participants with low literacy (42.9% had no formal or only primary education), questions were read aloud in Arabic, and responses were recorded verbatim by the interviewers. Interviewers received a 2-day training on neutral probing techniques (e.g., “Can you tell me more about that?”) to facilitate more detailed responses while avoiding bias. All responses were transcribed into a secure digital database; 10% of entries were double-checked for transcription accuracy.

### 2.5 Ethical considerations

The study was approved by the Ministry of health and Al-Ahliyya Amman University (AAU) ethical committees (IRB: AAU/1/6/2023-2024). Participants were informed about the study and provided written consent, ensuring voluntary participation and the right to withdraw. Anonymity was maintained with unique identifiers, and data were stored securely in a locked cabinet and password-protected database.

### 2.6 Statistical analysis

Data were analyzed using Prism 9.0 version, with descriptive statistics (means, SDs, frequencies, percentages) summarizing characteristics and scores. Bivariate analyses, including chi-square tests for categorical variables and independent t-tests for continuous variables, assessed associations between medication adherence (binary: ≥27 vs. ≤26) and factors such as cost, forgetting, and health literacy.

Multivariate logistic regression was performed to identify predictors of high medication adherence, adjusting for confounders including age, sex, income, education, insurance status, number of conditions, and number of medications, with adjusted odds ratios (AORs) and 95% confidence intervals reported. A significance level of p < 0.05 was adopted for all statistical tests. Missing data were assessed for each variable, and cases with incomplete key variables were excluded using complete case analysis. Overall, missing data were minimal (<5%) and did not warrant imputation.

Qualitative data were analyzed using Braun and Clarke’s six-phase thematic analysis framework, following an inductive coding approach. Two independent researchers analyzed responses in the original Arabic language using NVivo version 12, with emergent codes grouped into overarching themes. Discrepancies were resolved through discussion and consensus. Inter-coder reliability was calculated using Cohen’s kappa (κ = 0.85) to ensure analytic rigor. Thematic saturation was monitored throughout the coding process, with no new themes emerging after approximately 85% of responses had been analyzed, indicating adequate saturation. Themes were then translated into English for reporting and triangulated with quantitative findings (e.g., GMAS scores, income, rural residence) to enrich the interpretation of medication adherence patterns.

## 3 Results

### 3.1 Sociodemographic and clinical characteristics

A total of 420 patients with multimorbidity were recruited, 405 completed the survey (response rate: 96.4%). The sociodemographic characteristics of the participants are summarized in Table 1. Participants had a mean age of 58.3 years, with a near-even gender split. Most participants were married and from urban areas, and a significant proportion had low educational attainment and household income. The majority were insured (74.1%), although a quarter remained uninsured.

**TABLE 1:** Sociodemographic and clinical characteristics by medication adherence status (*N* = 405).

Variable	Total n (%)	Low adherence n (%)	High adherence n (%)	p-value
Age (years)				
18–30	35 (8.6)	13 (37.1)	22 (62.9)	0.028
31–45	80 (19.8)	34 (42.5)	46 (57.5)	
46–60	154 (38.0)	70 (45.5)	84 (54.5)	
≥61	136 (33.6)	50 (36.8)	86 (63.2)	
Gender				
Female	213 (52.6)	103 (48.4)	110 (51.6)	0.214
Male	192 (47.4)	82 (42.7)	110 (57.3)	
Marital status				
Single	42 (10.4)	16 (38.1)	26 (61.9)	0.682
Married	276 (68.1)	126 (45.7)	150 (54.3)	
Divorced	23 (5.7)	11 (47.8)	12 (52.2)	
Widowed	64 (15.8)	32 (50.0)	32 (50.0)	
Education				
No formal education	80 (19.8)	42 (52.5)	38 (47.5)	0.041
Primary school	102 (25.2)	50 (49.0)	52 (51.0)	
Secondary school	58 (14.3)	28 (48.3)	30 (51.7)	
Diploma/vocational	92 (22.7)	38 (41.3)	54 (58.7)	
University or higher	73 (18.0)	27 (37.0)	46 (63.0)	
Income (JOD)				
<300	117 (28.9)	65 (55.6)	52 (44.4)	<0.001
30–600	185 (45.7)	90 (48.6)	95 (51.4)	
601–1,200	75 (18.5)	25 (33.3)	50 (66.7)	
>1,200	28 (6.9)	5 (17.9)	23 (82.1)	
Residence				
Urban	248 (61.2)	103 (41.5)	145 (58.5)	0.008
Rural	157 (38.8)	82 (52.2)	75 (47.8)	
Employment status				
Employed	128 (31.6)	54 (42.2)	74 (57.8)	0.137
Unemployed	102 (25.2)	51 (50.0)	51 (50.0)	
Retired	145 (35.8)	62 (42.8)	83 (57.2)	
Other	30 (7.4)	18 (60.0)	12 (40.0)	
No. of conditions				
2	138 (34.1)	54 (39.1)	84 (60.9)	0.019
3	126 (31.1)	56 (44.4)	70 (55.6)	
4	87 (21.5)	41 (47.1)	46 (52.9)	
5	32 (7.9)	18 (56.3)	14 (43.8)	
≥6	22 (5.4)	16 (72.7)	6 (27.3)	
No. of Medications				
1	35 (8.6)	14 (40.0)	21 (60.0)	0.015
2–3	110 (27.2)	45 (40.9)	65 (59.1)	
4–6	154 (38.0)	65 (42.2)	89 (57.8)	
7–10	80 (19.8)	42 (52.5)	38 (47.5)	
>10	26 (6.4)	19 (73.1)	7 (26.9)	
Duration of use				
<1 year	41 (10.1)	18 (43.9)	23 (56.1)	0.312
1–5 years	132 (32.6)	58 (43.9)	74 (56.1)	
6–10 years	117 (28.9)	54 (46.2)	63 (53.8)	
>10 years	115 (28.4)	55 (47.8)	60 (52.2)	
Insurance				
No	105 (25.9)	58 (55.2)	47 (44.8)	0.003
Yes	300 (74.1)	127 (42.3)	173 (57.7)	
Insurance Type (*n* = 300)				
Public	120 (40.0)	54 (45.0)	66 (55.0)	0.011
Private	76 (25.3)	32 (42.1)	44 (57.9)	
Military	84 (28.0)	34 (40.5)	50 (59.5)	
Other	20 (6.7)	7 (35.0)	13 (65.0)	
Coverage (*n* = 300)				
Full	198 (66.0)	78 (39.4)	120 (60.6)	0.009
Partial	102 (34.0)	49 (48.0)	53 (52.0)	

Notes: High adherence = GMAS ≥27; Low adherence = GMAS ≤26. p-values from chi-square tests.

Participants reported a mean of 3.4 chronic conditions. Hypertension (72.3%) and diabetes (58.0%) were the most prevalent, followed by thyroid disease, hyperlipidemia, and arthritis (Table 2). The most commonly reported limitations in daily activity were due to arthritis, while most conditions showed high treatment and moderate control rates.

**TABLE 2:** Chronic conditions and medication adherence (*N* = 405).

Condition	Have it n (%)	Low adherence n (%)	High adherence n (%)	Treated n (%) adherent	Limits n (%) adherent	Controlled n (%) adherent	p-value
Diabetes	235 (58.0)	108 (46.0)	127 (54.0)	123/227 (54.2)	14/30 (46.7)	104/188 (55.3)	0.918
Hypertension	293 (72.3)	135 (46.1)	158 (53.9)	152/278 (54.7)	10/20 (50.0)	134/245 (54.7)	0.683
Heart disease	84 (20.7)	38 (45.2)	46 (54.8)	44/80 (55.0)	9/19 (47.4)	36/66 (54.5)	0.946
Asthma	41 (10.1)	19 (46.3)	22 (53.7)	20/37 (54.1)	4/9 (44.4)	18/32 (56.3)	0.927
COPD	25 (6.2)	12 (48.0)	13 (52.0)	11/22 (50.0)	3/6 (50.0)	10/19 (52.6)	0.804
Arthritis	91 (22.5)	48 (52.7)	43 (47.3)	35/69 (50.7)	16/38 (42.1)	25/50 (50.0)	0.099
Kidney disease	46 (11.4)	26 (56.5)	20 (43.5)	18/42 (42.9)	5/12 (41.7)	16/35 (45.7)	0.087
Depression/anxiety	35 (8.6)	18 (51.4)	17 (48.6)	13/26 (50.0)	3/7 (42.9)	10/20 (50.0)	0.467
Thyroid disease	128 (31.6)	58 (45.3)	70 (54.7)	63/115 (54.8)	9/18 (50.0)	51/92 (55.4)	0.941
High cholesterol	109 (26.9)	49 (45.0)	60 (55.0)	55/100 (55.0)	2/5 (40.0)	51/92 (55.4)	0.875
Lung disease (other)	18 (4.4)	9 (50.0)	9 (50.0)	8/15 (53.3)	1/3 (33.3)	7/13 (53.8)	0.678
Cancer	15 (3.7)	8 (53.3)	7 (46.7)	6/12 (50.0)	2/5 (40.0)	5/9 (55.6)	0.513
Other	36 (8.9)	17 (47.2)	19 (52.8)	15/28 (53.6)	3/7 (42.9)	13/24 (54.2)	0.834

Notes: Values presented as n (%) except in the “Treated,” “Limits,” and “Controlled” columns, which are shown as n/N (%), where n = number adherent and N = total with the condition. High adherence = GMAS ≥27; Low adherence = GMAS ≤26. p-values from chi-square tests.

The median number of medications taken daily was 5 (IQR: 3–7). Most participants had been using medications for more than 1 year. Medication adherence, defined as GMAS ≥27, was observed in 54.3% of the sample. Higher medication adherence was significantly associated with older age (p = 0.028), higher education (p = 0.041), greater income (p < 0.001), urban residence (p = 0.008), fewer chronic conditions (p = 0.019), lower pill burden (p = 0.015), and having insurance (p = 0.003), as detailed in Table 1. These stratified relationships are also illustrated in Table 5, showing differences in adherence based on pill count, income level, and urban versus rural residence.

Subgroup analysis revealed compounded effects of social and clinical factors. For instance, rural participants with income <300 JOD had lower medication adherence (38.5%) compared to urban participants with income >600 JOD (55.0%, p = 0.012). Similarly, females with ≥4 conditions were less adherent than males with only two conditions (45.0% vs. 58.3%, p = 0.025). Poor medication adherence was also linked to uncontrolled conditions such as arthritis and untreated hypertension.

### 3.2 Medication adherence

The mean GMAS score across the sample was 25.8 (SD = 5.6), reflecting moderate overall medication adherence (Table 3). Just over half of participants (54.3%) achieved high or good adherence (GMAS ≥27), while 42.0% showed partial adherence and a small minority (3.7%) reported low adherence (≤10).

**TABLE 3:** GMAS total and domain scores by medication adherence category (N = 405).

Domain/Item	Total reporting barrier n (%)	High (n = 120) n (%)	Good (n = 100) n (%)	Partial (n = 170) n (%)	Low (n = 15) n (%)	p-value
Patient behavior (Mean = 9.8/12)						
Forgetting	177 (43.7)	98 (81.7)	82 (82.0)	48 (28.2)	6 (40.0)	<0.001
Stop when feeling better	102 (25.2)	108 (90.0)	85 (85.0)	95 (55.9)	8 (53.3)	<0.001
Stop when feeling worse	85 (21.0)	110 (91.7)	88 (88.0)	105 (61.8)	10 (66.7)	<0.001
Take at different time	91 (22.5)	105 (87.5)	84 (84.0)	102 (60.0)	9 (60.0)	<0.001
Comorbidity/Pill Burden (Mean = 7.9/12)						
Busy schedule/higher pill burden	154 (38.0)	84 (70.0)	70 (70.0)	85 (50.0)	5 (33.3)	<0.001
Hard to swallow/multiple doses	118 (29.1)	92 (76.7)	75 (75.0)	90 (52.9)	6 (40.0)	<0.001
Avoid due to side effects	91 (22.5)	100 (83.3)	80 (80.0)	105 (61.8)	8 (53.3)	<0.001
Too many health problems	105 (25.9)	95 (79.2)	78 (78.0)	95 (55.9)	7 (46.7)	<0.001
Cost-Related (Mean = 8.1/9)						
Skip due to expense	214 (52.8)	90 (75.0)	76 (76.0)	65 (38.2)	3 (20.0)	<0.001
Avoid refilling due to cost	162 (40.0)	95 (79.2)	80 (80.0)	80 (47.1)	4 (26.7)	<0.001
Take smaller dose to save money	75 (18.5)	105 (87.5)	85 (85.0)	120 (70.6)	10 (66.7)	0.002
Total GMAS Score						
Mean (SD)	25.8 (5.6)	31.2 (1.1)	27.8 (0.9)	22.3 (3.8)	8.5 (2.1)	<0.001

Notes: *“Total Reporting Barrier” indicates participants reporting “Mostly” or “Sometimes.” Adherence category columns indicate the number and percentage of participants within each group reporting “Never” or “Rarely” experiencing the barrier, reflecting lower presence of that barrier. High adherence = GMAS ≥27; low adherence = GMAS ≤26. *p*-values calculated using chi-square tests.

Among GMAS domains, patient behavior and cost-related barriers were the most commonly reported challenges. Forgetfulness and busy schedules were particularly common among those with lower adherence to medications. Cost-related non-adherence—such as skipping doses due to medication expense—was substantially more prevalent among partial and low adherers. In contrast, participants with high adherence were significantly less likely to skip or delay medications across all domains (p < 0.001).

Correlation analysis showed that higher medication adherence was positively associated with income (r = 0.28, p < 0.001), and inversely related to the number of chronic conditions (r = −0.22, p = 0.001) and pill burden taken (r = −0.19, p = 0.004), indicating that financial and treatment burden significantly impact medication adherence patterns.

### 3.3 Health literacy

Health literacy significantly influenced medication adherence, as shown in Table 4. Participants with higher medication adherence (GMAS ≥27) were more confident in understanding medication labels, less likely to require assistance with medication instructions, and more knowledgeable about the purpose of their medications (p < 0.001 for all comparisons).

**TABLE 4:** Health Literacy Characteristics and Medication adherence (N = 405).

Variable	Total n (%)	Low adherence n (%)	High adherence n (%)	p-value
Confidence in understanding labels				
Very confident	167 (41.2)	54 (32.3)	113 (67.7)	<0.001
Somewhat confident	155 (38.3)	73 (47.1)	82 (52.9)	
Not at all confident	83 (20.5)	58 (69.9)	25 (30.1)	
Needing help to understand meds				
Never	245 (60.5)	103 (42.0)	142 (58.0)	0.002
Sometimes	128 (31.6)	64 (50.0)	64 (50.0)	
Always	32 (7.9)	18 (56.3)	14 (43.8)	
Knowing medication purposes				
Yes, know all	190 (46.9)	60 (31.6)	130 (68.4)	<0.001
Yes, know some	157 (38.8)	80 (51.0)	77 (49.0)	
Do not know	58 (14.3)	45 (77.6)	13 (22.4)	
Ease of asking questions				
Very easy	213 (52.6)	77 (36.2)	136 (63.8)	0.001
Somewhat easy	140 (34.6)	71 (50.7)	69 (49.3)	
Not easy	52 (12.8)	37 (71.2)	15 (28.8)	

Notes: High adherence = GMAS ≥27; Low adherence = GMAS ≤26. p-values from chi-square tests.

Ease of communication with healthcare providers was also associated with medication adherence; those who found it very easy to ask questions were more likely to be adherent. Conversely, these challenges were more prevalent among individuals with lower education, rural residence, and lower income. Overall, health literacy scores positively correlated with medication adherence (r = 0.35, p < 0.001), especially knowledge of medication purpose (r = 0.42, p < 0.001).

### 3.4 Barriers and facilitators to medication adherence

Barriers and facilitators were explored through bivariate and multivariate analyses, with results summarized in Table 5. The most common barriers reported among lower medication adherence categories included medication cost (52.8%), forgetfulness (43.7%), and pill burden (38.0%). These factors were significantly associated with reduced odds of medication adherence (p < 0.01 for all), especially among those with partial medication adherence, who frequently cited financial and memory-related challenges.

**TABLE 5:** Barriers and Facilitators to Medication adherence (N = 405).

Variable	Total n (%)	Low adherence n (%)	High adherence n (%)	p-value	AOR (95% CI)
Barriers					
Cost of medications	214 (52.8)	141 (65.9)	73 (34.1)	<0.001	0.42 (0.28–0.63)**
Forgetting	177 (43.7)	122 (68.9)	55 (31.1)	<0.001	0.38 (0.25–0.58)**
Higher pill burden	154 (38.0)	96 (62.3)	58 (37.7)	<0.001	0.55 (0.36–0.84)**
Transportation issues	117 (28.9)	69 (59.0)	48 (41.0)	0.001	0.61 (0.39–0.95)*
Difficulty getting to pharmacy	102 (25.2)	60 (58.8)	42 (41.2)	0.002	0.67 (0.42–1.07)
Side effects	91 (22.5)	56 (61.5)	35 (38.5)	0.004	0.70 (0.43–1.14)
Not believing medications help	64 (15.8)	45 (70.3)	19 (29.7)	<0.001	0.35 (0.19–0.65)**
Cultural/religious beliefs	40 (9.9)	26 (65.0)	14 (35.0)	0.027	0.58 (0.29–1.17)
Other	22 (5.4)	12 (54.5)	10 (45.5)	0.312	0.82 (0.33–2.04)
Facilitators					
Reminders	201 (49.6)	69 (34.3)	132 (65.7)	<0.001	2.45 (1.65–3.64)**
Support from family/friends	179 (44.2)	67 (37.4)	112 (62.6)	0.003	1.88 (1.24–2.85)**
Clear instructions from doctor	160 (39.5)	62 (38.8)	98 (61.3)	0.005	1.72 (1.13–2.62)**
Feeling better on medications	135 (33.3)	54 (40.0)	81 (60.0)	0.045	1.49 (0.97–2.29)*
Affordable cost	110 (27.2)	42 (38.2)	68 (61.8)	0.018	1.65 (1.04–2.61)*
Religious routines	92 (22.7)	32 (34.8)	60 (65.2)	0.030	1.78 (1.07–2.96)*
Other	15 (3.7)	7 (46.7)	8 (53.3)	0.872	1.12

Notes: High adherence = GMAS ≥27; Low adherence = GMAS ≤26. p-values from chi-square tests. AORs, from multiple logistic regression adjusted for age, sex, income, education, insurance, number of conditions, and medications. *p < 0.05, **p < 0.01.

The use of reminders (49.6%), family or social support (44.2%), and receiving clear medication instructions (39.5%) were significantly associated with higher medication adherence levels. Notably, the use of reminders showed the strongest positive association (AOR = 2.45, 95% CI [1.65–3.64], p < 0.01).

Perceived medication ineffectiveness and stopping treatment when feeling better were notable among low and partial adherers, reflecting intentional medication non-adherence. Although side effects were cited by 22.5% of participants, this factor did not retain significance in multivariate analysis. Overall, participants with partial medication adherence reported the most frequent modifiable barriers, suggesting they may benefit from tailored medication adherence support in future interventions.

### 3.5 Qualitative themes

Of the 405 participants, 287 (70.9%) provided qualitative responses, from which six major themes were identified (Table 6), primarily affecting those in the ‘partial’ medication adherence category. These themes were supported by multivariate analysis and corresponded with adherence levels as categorized by the four-tier GMAS breakdown.

**TABLE 6:** Thematic analysis of qualitative responses and illustrative quotes according to adherence category (*N* = 287).

Theme	n (%) of respondents	Example quote	Adherence correlation (p-value)	AOR (95% CI)	Proposed intervention
Economic Strain	52 (18.1)	“I skip doses to afford food”	p = 0.005 (vs. income)	0.48 (0.27–0.85)**	Subsidize medications for low-income patients (<300 JOD)
Access Gaps	34 (11.8)	“Pharmacies are far, transport unreliable”	p = 0.008 (vs. residence)	0.59 (0.31–1.12)	Deploy mobile pharmacies in rural areas
Cultural Distrust	25 (8.7)	“Herbs are safer than pills”	p = 0.027 (vs. beliefs)	0.52 (0.24–1.13)	Community education on medication efficacy
Religious Coping	29 (10.1)	“Prayer times remind me”	p = 0.030 (positive)	1.92 (1.03–3.58)*	Integrate pill schedules with prayer times
Caregiving Burden	41 (14.3)	“Family needs leave no time”	r = −0.31, p = 0.008	0.44 (0.25–0.78)**	Offer caregiver support programs (e.g., respite care)
Uncontrolled Symptoms	15 (5.2)	“Arthritis pain stops me”	p = 0.045 (vs. control)	0.39 (0.17–0.89)*	Enhance symptom management via specialist referrals

Notes: Themes were derived using thematic analysis from 287 qualitative responses. Categories reflect GMAS-assessed adherence levels. AORs, adjusted for age, gender, income, education, insurance, number of conditions, and medications. *p < 0.05, **p < 0.01.

Economic strain, reported by 54 participants, emerged as the most frequently cited barrier, with participants citing medication cost as a reason for medication non-adherence. This was particularly common among partial adherers, highlighting the potential utility of medication subsidies.

*“Sometimes I skip my medicine to afford rent or groceries.”* (Participant #112, partial medication adherence).

This illustrates how financial hardship can directly compete with medication adherence needs.

Access gaps were reported by 34 participants (11.8%), especially in rural areas, among those with unintentional medication non-adherence. Participants reported difficulties in accessing medications and healthcare providers, supporting mobile pharmacy outreach.

*“The clinic is too far. If my son cannot drive me, I just go without.”* (Participant #67, partial medication adherence).

This illustrates how logistical barriers in rural settings can impede consistent medication access.

Cultural distrust, reported by 25 participants (8.7%), reflected a preference for traditional or herbal remedies and skepticism toward conventional medications. Though not statistically significant, it highlights the importance of culturally sensitive health education.

*“I stopped the pills and used herbs because they feel more natural.”* (Participant #143, partial medication adherence).

This quote highlights the role of cultural beliefs in intentional non-adherence.

On the positive side, religious coping was reported by 29 participants (10.1%) and was associated with better medication adherence, especially among those aligning medication schedules with daily prayers.

*“I take my medicine after each prayer. It helps me remember.”* (Participant #21, good medication adherence).

This demonstrates how cultural and religious practices can facilitate adherence routines.

Caregiving burden, reported by 41 participants (14.3%), was frequently cited by women managing both family and health responsibilities. Busy schedules and forgetfulness were dominant challenges.

“*I’m taking care of my mother and kids. I forget about myself sometimes*.” (Participant #184, partial medication adherence).

This quote illustrates how caregiving demands can overshadow personal health management.

Lastly, uncontrolled symptoms, reported by 15 participants (5.2%), such as pain or side effects led some to avoid medications entirely. This finding points to a possible association with need for improved symptom management and greater access to specialist care.

“*I do not take my arthritis meds because they upset my stomach*.” (Participant #156, partial medication adherence).

This emphasizes how poorly managed side effects can lead to intentional medication non-adherence.

Together, these themes reinforce the importance of targeting partial adherers in future interventions and highlight practical, patient-informed solutions to improve medication adherence behavior.

## 4 Discussion

Medication adherence in chronic disease remains a global challenge, with only about half of patients in developed countries following long-term therapy recommendations on average (Al-Qasem et al., 2011). This problem is often exacerbated in low- and middle-income countries due to resource constraints and access barriers (Khoiry et al., 2023). Our finding of a 54.3% high/good adherence rate in Jordanian patients with multimorbidity aligns with this broader trend, indicating that nearly half of patients struggle with adherence to medications despite needing medications for multiple conditions. This rate is comparable to adherence levels reported in similar settings. For example, a study in Lebanon found 42.6% of chronic disease outpatients were adherent (Al-Hajje et al., 2015), and only 35.8% of Saudi patients with type 2 diabetes achieved high medication adherence (AlQarni et al., 2019). On the other hand, our adherence rate is lower than that reported in the UAE, where 78.6% of patients with multimorbidity were classified as highly adherent in a tertiary-care study. The relatively higher adherence in the UAE context has been attributed to stronger health system support and insurance coverage, underscoring how socioeconomic context can influence medication adherence (Allaham et al., 2022). Nonetheless, across the Middle East, medication adherence rates vary widely–a regional review noted estimates ranging from as low as 1.4% up to 88% in different disease contexts–but consistently point to suboptimal medication-taking in a significant fraction of patients (Al-Qasem et al., 2011). Our study contributes to this literature by providing recent data from Jordan and showing that, much like global patterns, medication adherence in a multimorbid population remains suboptimal.

Several barriers to adherence emerged from our study, mirroring factors reported globally and regionally. Economic hardship was a dominant theme: many patients struggled with medication costs, which is a well-recognized impediment to medication adherence in low- and middle-income countries (Rohatgi et al., 2021). Out-of-pocket medication expenses and lack of insurance were associated with lower adherence in our cohort, consistent with the findings from other Middle Eastern settings. For instance, a UAE study explicitly found that not having health insurance significantly lowered the odds of high adherence (Allaham et al., 2022). Similarly, in Saudi Arabia higher medication costs have been linked to poorer adherence (Fallatah et al., 2023). These findings suggest an association between financial barriers and patients skipping doses or rationing medications. Previous studies have also shown that medication adherence often improves markedly when cost barriers are removed or reduced, highlighting the importance of financial protection in chronic disease management (Allaham et al., 2022; Fallatah et al., 2023).

Another key barrier is treatment complexity and pill burden. Patients with multimorbidity typically manage several medications, and our results indicate that this pill burden was associated with lower medication adherence. This is consistent with evidence from across the region and beyond. A recent study in Jeddah found that a higher number of prescribed medications and more complex regimens were significant predictors of poorer medication adherence. In Jordan, polypharmacy increases medication errors, with each additional pre-admission medication raising the risk of discrepancies that complicate medication adherence (Abu Farha et al., 2021). Each additional pill adds to the “work” of managing a chronic illness, as patients must remember multiple dosing schedules and cope with potential side effects or drug interactions (Fallatah et al., 2023). Our findings reinforce that “pill burden” is not merely inconvenient but may be associated with missed doses and dropout from therapy. This has important implications for clinicians, who may consider simplifying regimens to help ease this burden on patients.

Beyond the numerical pill burden, the drug-specific burden of polypharmacy—including complex administration schedules, drug-drug interactions, and adverse effects—can further challenge adherence and may require targeted pharmacological consideration. Strategies such as medication reconciliation and deprescribing, particularly in older and multimorbid populations, have shown promise in reducing unnecessary medication use and improving adherence (Reeve et al., 2015). Additionally, intervention models like Medication Therapy Management (MTM) and clinical pharmacist-led adherence clinics have demonstrated effectiveness in supporting medication adherence and optimizing pharmacotherapy outcomes and could be adapted within the Jordanian healthcare system (Viswanathan et al., 2015; Mekonnen et al., 2016).

We also identified that rural residence was associated with lower medication adherence compared to urban residence. This urban–rural disparity has been similarly observed in other populations. Rural patients often face longer travel distances to healthcare facilities and pharmacies, limited availability of medications, and fewer healthcare resources, all of which can hinder consistent medication refills and follow-up (Syed et al., 2013). Similar patterns have been observed internationally; for example, a study in Taiwan found that most elderly patients in rural areas had poor medication adherence and low refill continuity compared to urban populations (Li et al., 2024).

Beyond these major barriers, our qualitative findings and the wider literature point to other factors that can undermine medication adherence in multimorbidity. Cognitive and psychological factors are important. For instance, forgetfulness and memory decline (especially in older patients) contribute to medication non-adherence, and comorbid depression or anxiety can reduce motivation to take medications (Kim and Kim, 2024). Misconceptions about medications also play a role; some patients harbour beliefs that higher pill burden are harmful or doubt the necessity of their treatment once symptoms abate (Rafhi et al., 2024).

Counterbalancing the barriers, we identified several facilitators that were associated with a higher medication adherence rate. Strong family support was commonly cited, where family members helped remind, procure, or administer medications. This is consistent with literature showing that social support is positively associated with medication adherence (Shahin et al., 2021).

The use of reminder systems (like alarms, pill boxes, or linking pills to daily habits) was also linked to better medication adherence in our study. Notably, some participants used prayer times as natural anchors for medication routines. While formal literature on prayer as a reminder is scant, the concept aligns with habit-formation strategies known to benefit medication adherence (Ghai et al., 2024).

Religious coping was another factor positively associated with medication adherence. Patients who framed their treatment as a religious responsibility or found strength in their faith reported better medication-taking behavior. Similar patterns have been noted in patients with cardiovascular disease, though outcomes may vary based on context (Elhag et al., 2022).

Health insurance coverage was also associated with higher medication adherence, likely through reducing medication costs and improving access to follow-up care. Prior studies in the UAE and other countries have shown that insured patients are more likely to refill prescriptions and attend scheduled clinic visits (Allaham et al., 2022).

These observed associations have important implications for health systems, clinical practice, and policy, particularly in the context of strengthening health services to meet the Sustainable Development Goals. Firstly, improving medication adherence among patients with multimorbidity is essential for achieving better health outcomes and optimizing healthcare utilization. From a health policy perspective, our study highlights several leverage points. The identification of cost and insurance as pivotal factors suggests that policies aimed at reducing financial barriers may help improve medication adherence. This could include expanding public insurance enrolment, ensuring essential medications for chronic diseases are subsidized or free at point of service (especially for low-income and rural patients), and regulating medicine prices. Strengthening the patient–provider relationship is another implication. Patients who are satisfied with their care and who trust their healthcare providers tend to adhere better. Training clinicians in effective counselling, empathy, and clear communication about medications may foster this trust and partnership in care.

In terms of the broader development goals, ensuring good health and wellbeing (SDG 3) for populations increasingly means tackling non-communicable diseases and their risk factors. Medication adherence is considered a central component in the management of NCDs (like diabetes, heart disease, chronic lung disease, etc.), which aligns with SDG 3.4 – the reduction of premature mortality from NCDs by one-third by 2030. Policymakers should recognize that medication non-adherence is a public health crisis in its own right, dampening the impact of medical advancements. Enhancing the effectiveness of therapy adherence interventions may yield population health benefits that rival those of new pharmacological innovations. In other words, the potential return on investment for medication adherence support is substantial, as it may lead to fewer complications, improved quality of life, and more efficient use of healthcare resources.

These findings have implications that extend beyond Jordan. Multimorbidity is a growing global challenge, and the lessons learned from our study can inform therapy adherence strategies in other settings. For instance, countries with similar cultural values, economic constraints, or healthcare system structures may benefit from adapting these insights to their own contexts. Even high-income countries with diverse cultural populations might find value in recognizing the role of family, faith, and financial support in improving therapy adherence. By addressing known barriers—such as costs, fragmented care, and lack of coordination—and building on facilitators like social support, insurance coverage, and cultural practices, health systems worldwide may be able to support improved medication adherence rates, reduce the burden of chronic diseases, and achieve better health outcomes.

Our findings also hold particular relevance for the Jordanian healthcare context. While many medication adherence barriers identified in this study—such as cost, polypharmacy, and forgetfulness—are common across Middle Eastern populations, certain characteristics may be more pronounced in Jordan. For example, limited public insurance coverage and a high reliance on out-of-pocket payments make economic barriers especially significant, in contrast to Gulf countries like the UAE or Saudi Arabia, where broader insurance coverage supports better medication access. Cultural facilitators, such as synchronizing medication intake with daily prayers, also appear more deliberately used among Jordanian patients, offering a culturally anchored strategy to improve medication adherence. Moreover, caregiving responsibilities—especially among women—reflect traditional family structures that may add to treatment burden and require tailored support. Finally, distrust in pharmaceuticals and the use of herbal remedies, particularly in rural areas, point to the need for stronger community-based education campaigns. Recognizing these sociocultural specificities is essential for designing locally effective medication adherence interventions and offers insight for similarly structured healthcare systems in the region.

## 5 Conclusion

This mixed-methods study revealed that only 54.3% of Jordanian patients with multimorbidity demonstrated high or good medication adherence (GMAS ≥27), with a mean score of 25.8 (SD = 5.6). Economic hardship, rural residence, and high pill burden were the most prominent barriers to adherence, particularly among those with partial adherence. In contrast, factors such as reminder systems and religious coping practices were positively associated with better adherence behavior. These findings underscore the urgent need for context-specific interventions—such as subsidized medication programs, mobile pharmacy services for underserved areas, and culturally tailored reminder strategies—to improve adherence and advance progress toward Sustainable Development Goal 3 in Jordan’s resource-constrained healthcare system.

The use of a validated adherence scale alongside qualitative patient insights enhances the study’s comprehensiveness, while data from multiple clinical sites improve its generalizability. However, given the cross-sectional nature of the study, future longitudinal and interventional research is essential to evaluate the long-term impact and causal effectiveness of these proposed strategies. Additionally, integrating pharmacists into multidisciplinary care teams may offer a promising avenue to support medication adherence through individualized counseling, medication reconciliation, and therapeutic monitoring. Strengthening such systems is critical not only to enhance therapeutic outcomes but also to reduce preventable pharmacologic risks—such as toxicity, antimicrobial resistance, and treatment failure—especially in populations with complex chronic conditions.

## 6 Limitations

This study is not free of limitations. First, the use of convenience sampling may introduce selection bias. This approach may have overrepresented patients who regularly attend tertiary hospital clinics, potentially skewing the sample toward those with better healthcare access, higher health literacy, or more stable disease management compared to the broader Jordanian multimorbid population. To mitigate these limitations in future studies, alternative sampling strategies could enhance representativeness. Stratified random sampling, for instance, could ensure proportional representation of key subgroups, such as rural versus urban residents, or income levels, which significantly influenced medication adherence. Additionally, cluster sampling across diverse healthcare settings (primary care clinics or community pharmacies) could capture patients less likely to access tertiary care, addressing potential underrepresentation of marginalized groups.

Second, while our study quantified polypharmacy using pill count, our dataset did not capture detailed pharmacological profiles, limiting analysis of pharmacological complexity in medication adherence. Future studies should consider collecting data on drug classes, potential drug-drug interactions, pharmacokinetic properties, and the use of high-risk or complex administration medications, as these factors may significantly influence adherence behaviour in multimorbid populations beyond the sheer number of medications taken. Additionally, our dataset did not capture medication classes, which limited the ability to analyze adherence differences across drug categories (e.g., antihypertensives versus hypoglycemics), a factor of pharmacological relevance that future studies should explore.

Third, as this study is cross-sectional, all observed associations are correlational. Causal inferences cannot be made from this design. Therefore, statements implying causality were avoided or rephrased, and future longitudinal and interventional research is needed to explore these relationships more definitively.

Forth, self-reported data, despite clinic record cross-verification, remain subject to recall bias, particularly for treatment, limitation, and control status in the comorbidity checklist.

## Data Availability

The original contributions presented in the study are included in the article/supplementary material, further inquiries can be directed to the corresponding author.
